# Paradoxical counteraction by imatinib against cell death in myeloid progenitor 32D cells expressing p210BCR-ABL

**DOI:** 10.18632/oncotarget.25849

**Published:** 2018-08-03

**Authors:** Morichika Takita, Fujiko Tsukahara, Taishi Mishima, Katsuaki Ieguchi, Masayuki Yamada, Hiroaki Honda, Yoshiro Maru

**Affiliations:** ^1^ Department of Pharmacology, Tokyo Women's Medical University, Tokyo, Japan; ^2^ Center for Medical Education, Graduate School of Medicine, Kyoto University, Kyoto, Japan; ^3^ Institute of Laboratory Animals, Tokyo Women's Medical University, Tokyo, Japan

**Keywords:** chronic myeloid leukemia, p210BCR-ABL, Imatinib, cell death, differentiation

## Abstract

Chronic myeloid leukemia (CML) is believed to be caused by the tyrosine kinase p210BCR-ABL, which exhibits growth-promoting and anti-apoptotic activities. However, mechanisms that allow cell differentiation in CML still remain elusive. Here we established tetracycline (Tet)-regulatable p210BCR-ABL-expressing murine 32D myeloid progenitor (32D/TetOff-p210) cells to explore p210BCR-ABL-induced cell death and differentiation. Tet-regulatable overexpression of p210BCR-ABL induced cell death due to the activation of both caspase-1 and caspase-3, coincident with the differentiation from myeloid progenitors into CD11b^+^Ly6C^+^Ly6G^+^ cells with segmented nuclei, exemplified as granulocytic myeloid-derived suppressor cells (G-MDSC), and the ability to secrete IL-1β, TNF-α, and S100A8/A9 into the culture supernatant. Treatment with imatinib almost completely abrogated all these phenotypes. Moreover, overexpression of a sensor of activated caspase-1 based on fluorescence resonance energy transfer (FRET) probe enabled us to detect activation of caspase-1 in a human CML cell line, K562. Furthermore, increased numbers of splenic G-MDSC associated with enhancement of S100A8/A9 production were observed in transgenic mice expressing p210BCR-ABL compared with that in wild-type mice. We also propose the novel mode of cell death in this 32D/TetOff-p210 system termed as myeloptosis.

## INTRODUCTION

Chronic myeloid leukemia (CML) is a hematopoietic malignant disease caused by the tyrosine kinase p210BCR-ABL. Although the most characteristic manifestation of CML is the lack of a differentiation block in leukemic cells, there has been almost no biological system in which cell differentiation followed by cell death occurs in a p210BCR-ABL-dependent manner [1–4]. A previous study has shown that interleukin-1β (IL-1β) is elevated in the bone marrow of BCR-ABL transgenic (TG) SCL-tTa/BCR-ABL mice [5], but the precise mechanism and the biological significance of BCR-ABL-induced IL-1β elevation is unclear. Caspase-1 was originally identified as an IL-1β converting enzyme, which converts proIL-1β to active IL-1β [6]. Multiprotein complexes called inflammasomes, such as NLRP3 (Nod-like receptor family, pyrin domain containing 3), are required for caspase-1 activation [7]. Caspase-1-induced cell death is called pyroptosis, and is accompanied by the secretion of proinflammatory cytokines, such as IL-1β and IL-18 [8]. Although detection of activated caspase-1 is often difficult, a sensor of activated caspase-1 based on fluorescence resonance energy transfer (FRET) probe (SCAT1) has recently been developed and shown to be highly sensitive and specific to visualize caspase-1 activity even in a single cell [9].

Myeloid derived suppressor cells (MDSC) suppress immune responses against tumor antigens and promote cancer metastasis [10]. MDSC possess potent immune suppressor functions as initially described in tumor-bearing mice models [11]. Murine MDSC are subdivided into granulocytic MDSC (G-MDSC; CD11b^+^Ly6C^low^Ly6G^+^ cells) and monocytic MDSC (M-MDSC; CD11b^+^Ly6C^high^Ly6G^-^ cells), and human MDSC are subdivided into G-MDSC (CD11b^+^CD33^+^HLA-DR^neg/low^CD14^-^ cells) and M-MDSC (CD11b^+^CD33^+^HLA-DR^neg/low^ CD14^+^ cells) [10]. A recent clinical study has shown that G-MDSC numbers were increased significantly in Sokal high risk patients with CML compared with those in control subjects [12].

Tyrosine kinase activities are required for cell proliferation and differentiation. A previous study has shown that retroviral expression of v-*src* induces neuronal differentiation in the culture system of PC12 rat phaeochromocytoma cells [13]. Furthermore, retroviral infection with p210BCR-ABL in bone marrow-derived multipotent hematopoietic progenitors stimulates cell growth and differentiation into mast cells, macrophages, granulocytes, and B lymphoids in the soft agar colony assay [1].

In the present study, we established tetracycline (Tet)-regulatable p210BCR-ABL-expressing 32D myeloid progenitor (32D/TetOff-p210) cells of murine origin to explore p210BCR-ABL-induced cell death and differentiation. We found that Tet-regulatable overexpression of p210BCR-ABL-induced cell death was caused by caspase-1 and -3 activations, coincident with the differentiation from myeloid progenitors into G-MDSC and the secretion of IL-1β, tumor necrosis factor-α (TNF-α), and S100A8/A9 in 32D/TetOff-p210 cells. Furthermore, increased numbers of G-MDSC associated with enhancement of S100A8/A9 production were observed in TG mice expressing p210BCR-ABL compared with those in wild-type (WT) mice. Here we propose a novel mode of cell death, termed as myeloptosis, induced by Tet-regulatable overexpression of p210BCR-ABL in 32D/TetOff-p210 cells.

## RESULTS

### Influence of p210BCR-ABL overexpression on caspase-1 activation

**Figure 1:**
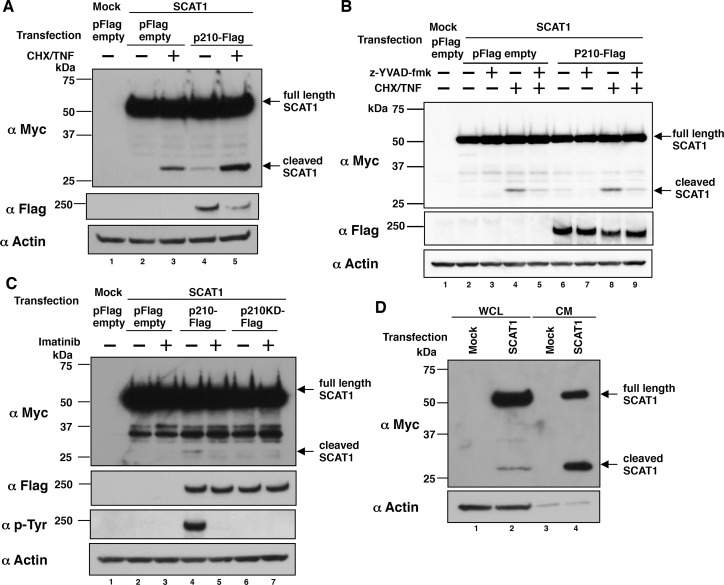
p210BCR-ABL-induced SCAT1 cleavage is dependent on both activities of BCR-ABL tyrosine kinase and caspase-1 **(A)** HeLa cells were transiently transfected with SCAT1 and Flag-tagged p210BCR-ABL (p210-Flag). At 43 h after transfection, cells were washed with PBS and then treated with serum-free DMEM in the presence or absence of TNF-α (50 ng/ml) and cycloheximide (CHX) (10 μg/ml) for 5 h. WCL were prepared and subjected to immunoblotting. Bands were visualized by probing with antibodies against Myc tag or Flag tag or actin. **(B)** HeLa cells were transiently transfected with SCAT1 and p210-Flag or pFlag empty vector. At 24 h after transfection, z-YVAD-fmk (20 μM) was added and further cultured for 19 h; cells were washed with PBS and then treated with serum-free DMEM with or without z-YVAD-fmk (20 μM) and/or TNF-α (50 ng/ml) and cycloheximide (10 μg/ml) for 5 h. WCL were prepared and subjected to immunoblotting. Bands were visualized by probing with antibodies against Myc tag, Flag tag or actin. **(C)** HeLa cells were transiently transfected with SCAT1 and p210-Flag or a Flag-tagged kinase-dead mutant of p210BCR-ABL (p210KD-Flag) or pFlag empty vector. At 24 h after transfection, imatinib (10 μM) was added and further cultured for 19 h, cells were washed with PBS and then treated with serum-free DMEM with or without imatinib (10 μM) for 5 h. WCL were prepared and subjected to immunoblotting. Bands were visualized by probing with antibodies against Myc tag, Flag tag, phospho Tyr (p-Tyr) or actin. **(D)** K562 cells were transiently transfected with SCAT1 cDNA. At 43 h after transfection, cells were washed with PBS and then subjected to medium change to serum-free RPMI1640 for 5 h. WCL and conditioned medium were prepared and subjected to immunoblotting. Bands were visualized by probing with antibodies against Myc tag or actin.

To clarify the involvement of p210BCR-ABL in caspase-1 activation, we first induced overexpression of both p210BCR-ABL and SCAT1 [9], and monitored SCAT1 cleavage in HeLa cells. Because SCAT1 harbors the caspase-1 cleavage site YVAD in the linker region, it can be recognized by activated caspase-1 and its cleavage reflects caspase-1 activation [9]. SCAT1 was detected as a full-length form, an approximately 50-kDa band probed with anti-Myc antibody, in HeLa cells transfected only with SCAT1 cDNA (Figure 1A, lane 2). When the cells were treated with a combination of cycloheximide and TNF-α (CHX/TNF), which can induce caspase activation and cell death [14], the cleaved SCAT1 was detected as an approximately 27-kDa band (Figure 1A, lane 3). The co-expression of Flag-tagged wild type p210BCR-ABL (p210-Flag) and SCAT1 weakly but substantially promoted SCAT1 cleavage, which was enhanced by 9.2-fold when additionally treated with CHX/TNF (Figure 1A, lanes 4 and 5). Treatment with a caspase-1 specific inhibitor, z-YVAD-fmk, inhibited the SCAT1 cleavage in cells co-transfected with SCAT1 and p210-Flag in the presence or absence of CHX/TNF (Figure 1B, lanes 6 vs 7, lanes 8 vs 9). Treatment with a BCR-ABL tyrosine kinase inhibitor, imatinib, inhibited both SCAT1 cleavage and tyrosine phosphorylation of p210-Flag (Figure 1C, lanes 4 vs 5). Furthermore, we could barely detect SCAT1 cleavage in cells transfected with a Flag-tagged kinase-dead mutant of p210BCR-ABL (p210KD-Flag) compared with the p210-Flag (Figure 1C, lanes 4 vs 6). These results suggest p210BCR-ABL-induced SCAT1 cleavage is dependent on both activities of BCR-ABL tyrosine kinase and caspase-1.

To demonstrate p210BCR-ABL-induced caspase-1 activation in a CML cell line, we subsequently tested whether SCAT1 cleavage can be detected in K562, a human CML cell line, which constitutively expresses p210BCR-ABL. SCAT1 cleavage was observed in whole cell lysate (WCL) and in the cell culture supernatant of K562 cells transfected with SCAT1 cDNA (Figure 1D, lanes 2 and 4). These results suggest p210BCR-ABL likely induces caspase-1 activation in K562 cells.

### Establishment of Tet-regulatable p210BCR-ABL-expressing mouse myeloid progenitor 32D cells

**Figure 2:**
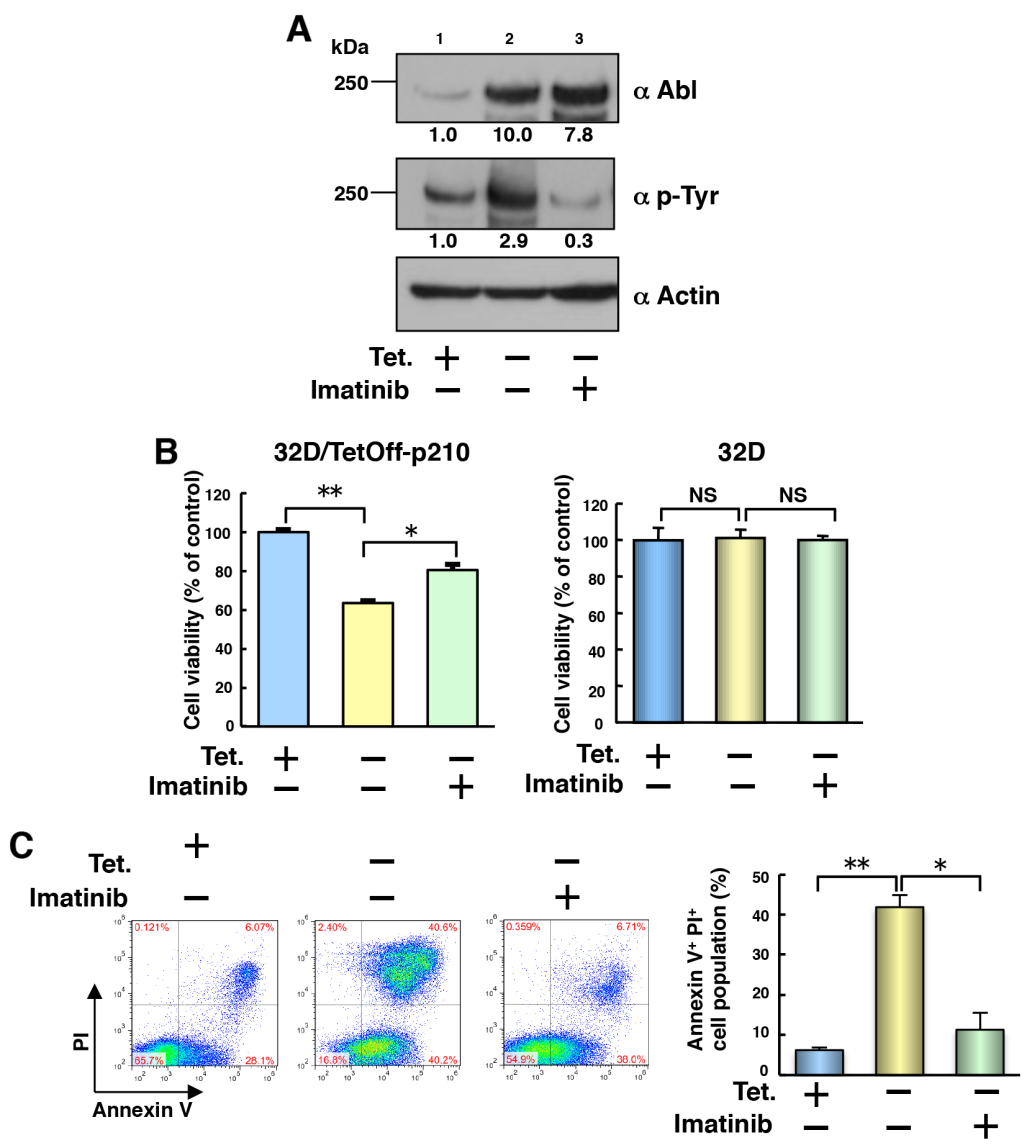
Tet-regulatable overexpression of p210BCR-ABL induces cell death in 32D/TetOff-p210 cells **(A)** 32D/TetOff-p210 cells were Tet-supplied or depleted and then cultured for 48 h in the presence or absence of imatinib (1 μM). WCL were subjected to immunoblotting. The values of relative band intensity versus the Tet (+) control were shown below each panel. Data are representative of three independent experiments. **(B)** 32D/TetOff-p210 cells or parental 32D cells were Tet-supplied or depleted and then cultured with IL-3 supplement for 48 h in the presence or absence of imatinib (1 μM). Cell viability was determined by WST-8 assay. ^*^P < 0.01, ^**^P < 0.001. Data are shown as mean ± SEM (*n* = 6) and are representative of three independent experiments. NS indicates no significant difference. **(C)** 32D/TetOff-p210 cells were Tet-supplied or depleted and then cultured for 96 h in the presence or absence of imatinib (1 μM). Cells were double-stained with annexin V and PI and analyzed by flow cytometry. The proportion of cell population with the representative data is shown in each panel. Four independent double-staining experiments were performed and statistical analysis was executed. ^*^P < 0.01, ^**^P < 0.001. Data are shown as mean ± SEM (*n* = 4).

Having shown that p210BCR-ABL potentially activated caspase-1 in a CML cell line (Figure 1D), we then established a Tet-regulatable p210BCR-ABL-expressing mouse myeloid cell line using the method previously reported [15, 16]. Because a previous study has shown that mouse myeloid progenitor 32D cells differentiate into granulocytes by treatment with granulocyte-colony stimulation factor (G-CSF) [17], we selected 32D cells to establish Tet-depletion-induced p210BCR-ABL-expressing (32D/TetOff-p210) cells and examined p210BCR-ABL-induced cell death and differentiation. p210BCR-ABL protein was induced by 10-fold under Tet-depletion (Figure 2A, lanes 1 vs 2). Treatment with imatinib decreased tyrosine-phosphorylated p210BCR-ABL but not total p210BCR-ABL protein amounts in 32D/TetOff-p210 cells (Figure 2A, lanes 2 vs 3). Leaky expression of p210BCR-ABL protein was also detected in the presence of Tet (Figure 2A, lane1). The level of p210BCR-ABL protein in Tet-depleted 32D/TetOff-p210 cells was 7-fold higher than that of Ba/F3 cells stably expressing p210-Flag (Supplementary Figure 1). IL-3 supplement was required for cell growth in parental 32D cells but not in 32D/TetOff-p210 cells because leaky expression of p210BCR-ABL contributed to cell growth.

### Effect of Tet-regulatable overexpression of p210BCR-ABL on cell viability and cell death in 32D/TetOff-p210 cells

To examine the influence of Tet-regulatable overexpression of p210BCR-ABL on cell growth, we performed mitochondrion dehydrogenase-based colorimetric cell proliferation WST-8 [2-(2-methoxy-4-nitrophenyl)-3-(4-nitrophenyl)-5-(2,4-disulfophenyl)-2H-tetrazolium, monosodium salt] assay in 32D/TetOff-p210 cells. Cell viability decreased markedly in Tet-depleted 32D/TetOff-p210 cells compared with that in Tet-supplied 32D/TetOff-p210 cells and was partially, but significantly, restored by treatment with imatinib (Figure 2B, left). Tet and/or imatinib did not remotely affect cell viability in the parental 32D cells (Figure 2B, right). These results suggest that Tet-regulatable overexpression of p210BCR-ABL may decrease cell viability in a BCR-ABL tyrosine kinase activity-dependent manner in 32D/TetOff-p210 cells. p210BCR-ABL-induced decrease in cell viability observed in 32D/TetOff-p210 cells was unexpected because previous studies have shown that p210BCR-ABL activates phosphatidylinositol 3 kinase (PI3K)-Akt pathway to enhance cell growth [18] and Bcl-X transcription, through signal transducer and activator of transcription 5 (STAT5), to inhibit apoptosis in CML cells [19].

To examine the cause of decreased cell viability in 32D/TetOff-p210 cells, we next analyzed the mode of cell death by double staining using annexin V and propidium iodide (PI). p210BCR-ABL expression by Tet-depletion significantly increased the annexin V^+^/PI^+^ cell population by 40.6% compared with that in Tet-supplied (6.07%) 32D/TetOff-p210 cells and it was significantly restored by 6.71% in the presence of imatinib (Figure 2C). Furthermore, neither a necrosis inhibitor (IM-54) nor an apoptosis inhibitor (Q-VD-OPH) prevented p210BCR-ABL-induced cell death in Tet-depleted 32D/TetOff-p210 cells (Supplementary Figure 2). These results suggest that p210BCR-ABL-induced cell death is neither apoptosis [20] nor necrosis [21].

### Tet-regulatable overexpression of p210BCR-ABL activates both caspase-1 and caspase-3 in 32D/TetOff-p210 cells

**Figure 3:**
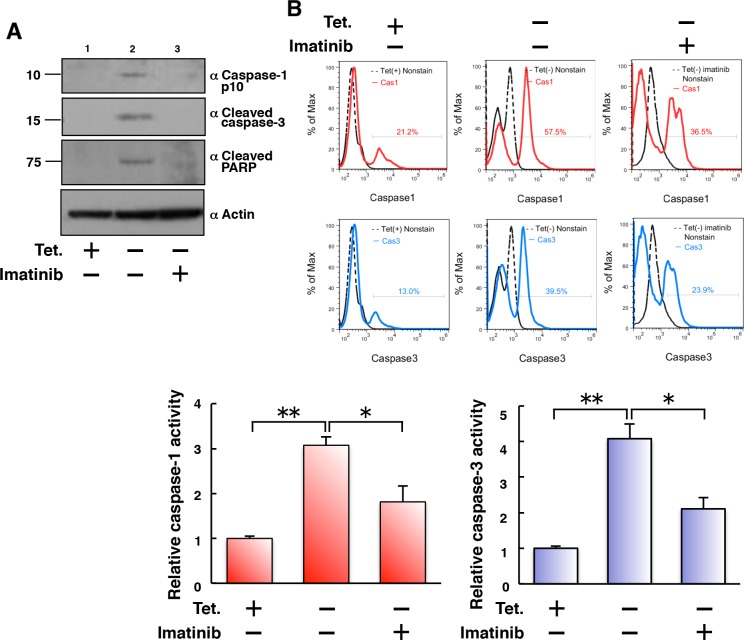
Tet-regulatable overexpression of p210BCR-ABL activates both caspase-1 and caspase-3 in 32D/TetOff-p210 cells **(A)** 32D/TetOff-p210 cells were Tet-depleted and then cultured for 48 h. WCL were subjected to immunoblotting. Bands were visualized by probing with antibodies against caspase-1 p10, cleaved caspase-3 or cleaved PARP. **(B)** 32D/TetOff-p210 cells were Tet-depleted or supplied and then cultured in the presence or absence of imatinib (1 μM) for 96 h and then incubated with FLICA 660 active caspase-1 or caspase-3 detection probe and analyzed by flow cytometry. The merged histogram (solid line) with non-stain control (dashed line) is shown in each panel. The proportion of FLICA-positive cell population max is shown in each panel. Three independent FLICA caspase-1/3 assays were performed, and statistical analysis was executed, as shown in lower graphs. ^*^P < 0.05, ^**^P < 0.01. Data are shown as mean ± SEM (*n* = 3).

We then examined caspases as effectors of cell death in 32D/TetOff-p210 cells. Tet-regulatable overexpression of p210BCR-ABL by Tet-depletion induced expression of caspase-1 p10, cleaved caspase-3, and cleaved poly ADP ribose polymerase (PARP) (Figure 3A, lane 2), which was attenuated by the presence of imatinib in 32D/TetOff-p210 cells (Figure 3A, lane 3). FLICA (fluorescently labeled inhibitor of caspases), which is a non-cytotoxic and cell-permeable reagent for detection of activated caspases [22], showed that Tet-regulatable overexpression of p210BCR-ABL significantly activated caspase-1 and caspase-3. Moreover, activated caspase-1 and caspase-3 were partially but significantly attenuated by the presence of imatinib in 32D/TetOff-p210 cells (Figure 3B). These results suggest that Tet-regulatable overexpression of p210BCR-ABL confers the combined feature of both activated caspase-1-induced pyroptosis and activated capase-3-induced apoptosis on 32D/TetOff-p210 cells. Furthermore, we tested whether glibenclamide, a NLRP3 inflammasome inhibitor, can prevent the activation of both caspase-1 and caspase-3 induced by Tet-regulatable p210BCR-ABL expression in 32D/TetOff-p210 cells. Glibenclamide did not prevent the activation of both caspase-1 and caspase-3 (Supplementary Figure 3). These results suggest that NLRP3 may not be involved in the activation of both caspase-1 and caspase-3 in 32D/TetOff-p210 cells.

### Induced production of proinflammatory cytokines by Tet-regulatable overexpression of p210BCR-ABL in 32D/TetOff-p210 cells and enhanced production of S100A8/A9 in BCR-ABL TG mice

**Figure 4:**
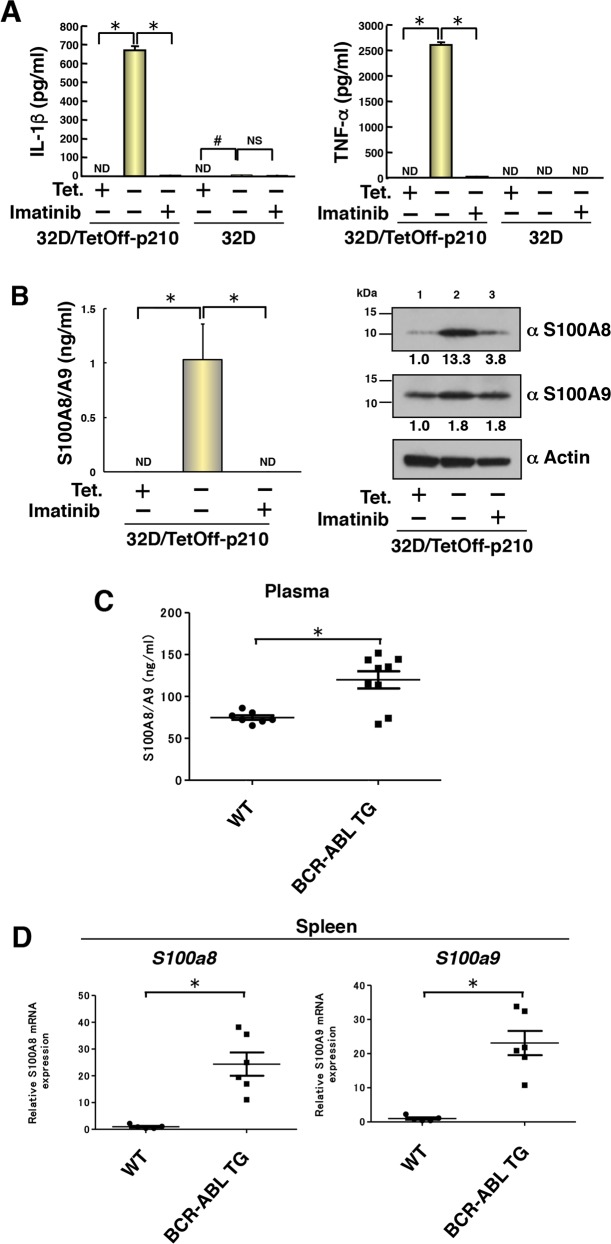
Induced production of proinflammatory cytokines by Tet-regulatable overexpression of p210BCR-ABL in 32D/TetOff-p210 cells and enhanced production of S100A8/A9 in BCR-ABL TG mice 32D/TetOff-p210 cells or parental 32D cells were Tet-supplied or depleted and then cultured with IL-3 supplement for 96 h to collect the culture supernatant and WCL. **(A)** ELISA was performed to determine concentration of IL-1β and TNF-α in the culture supernatant. ^*^P < 0.001, ^#^*P* < 0.05. Data are shown as mean ± SEM (*n* = 3) and are representative of three independent experiments. ND indicates not detected (under the detection limit). NS indicates no significant difference. **(B)** ELISA was performed to determine concentration of S100A8/A9 in the culture supernatant. ^*^P < 0.05. Data are shown as mean ± SEM (*n* = 3). ND indicates not detected (under the detection limit). WCL were subjected to immunoblotting. Bands were visualized by probing with antibodies against S100A8, S100A9 or actin. The values of relative band intensity versus the Tet (+) control are shown under each panel. **(C)** ELISA was performed to determine concentration of S100A8/A9 in the plasma of 8-month-old WT (*n* = 7) and BCR-ABL TG (*n* = 9) mice. Data are shown as mean ± SEM. ^*^P < 0.05. **(D)** Expressions of both *S100a8* and *S100a9* in the spleen of 8-month-old WT (*n* = 5) and BCR-ABL TG (*n* = 6) mice were determined by quantitative RT-PCR. Data are shown as mean ± SEM. ^*^P < 0.005. Statistical significance was evaluated by Mann–Whitney *U* test.

Caspase-1 activation leads to the secretion of proinflammatory cytokines [6], and CML stem cells produce a higher level of TNF-α than that by normal counterparts, as described previously [23]. Therefore, we measured the concentration of IL-1β and TNF-α in the culture supernatant of 32D/TetOff-p210 cells. Tet-regulatable overexpression of p210BCR-ABL by Tet-depletion significantly induced the secretion of IL-1β and TNF-α into the culture supernatant, which was almost completely inhibited by the treatment with imatinib in 32D/TetOff-p210 cells (Figure 4A). However, the secretion of these cytokines was not observed in parental 32D cells (Figure 4A). These results suggest that p210BCR-ABL-induced cell death is similar to pyroptosis [24]. To investigate the biological function of TNF-α and IL-1β in inducing cell death, we tested the effect of blocking antibodies against cell death of 32D/TetOff-p210 cells. Neither blocking antibody against TNF-α nor IL-1β prevented p210BCR-ABL-induced cell death in 32D/TetOff-p210 cells (Supplementary Figure 4). These data suggests that p210BCR-ABL-induced cell death is independent of both activities of TNF-α and IL-1β.

S100A8 and S100A9 are induced by primary tumor and chemoattract CD11b^+^ bone marrow-derived cells in pre-metastatic lungs, as reported previously by us [25]. A recent study has shown that S100A8 and S100A9 induce the secretion of cytokines, such as IL-1β, IL-6, IL-8, and increase the inflammasome NLRP3 via reactive oxygen species (ROS)-dependent activation of nuclear factor-κB (NF-κB) in human peripheral blood mononuclear cells [26]. Interestingly, Tet-regulatable overexpression of p210BCR-ABL by Tet-depletion significantly induced the secretion of S100A8/A9 heterodimer into the cell culture supernatant, which was also completely inhibited by treatment with imatinib in 32D/TetOff-p210 cells (Figure 4B, left). Furthermore, Tet-regulatable overexpression of p210BCR-ABL by Tet-depletion also induced S100A8 proteins by 13.3-fold and S100A9 by 1.8-fold in WCL of 32D/TetOff-p210 cells (Figure 4B, right, lanes 1 vs 2). Imatinib inhibited the upregulation of S100A8 but not S100A9 in WCL of 32D/TetOff-p210 cells (Figure 4B, right, lane 3). These results suggest that S100A8, but not S100A9, was primarily induced by Tet-regulatable overexpression of p210BCR-ABL in 32D/TetOff-p210 cells and may participate in the regulation of inflammasome. To examine the level of S100A8/A9 production in CML *in vivo*, we analyzed BCR-ABL TG mice [27] as a human CML model. In ELISA assay, plasma S100A8/A9 level was significantly induced in BCR-ABL TG mice compared with that in wild-type (WT) mice (Figure 4C). Furthermore, mRNA expressions of both *S100a8* and *S100a9* in the spleen were significantly enhanced in BCR-ABL TG mice compared with those in WT mice (Figure 4D). These results suggest that S100A8/A9 may participate in the progression of CML *in vivo*.

### Tet-regulatable overexpression of p210BCR-ABL induces differentiation from myeloid progenitor cells into G-MDSC in 32D/TetOff-p210 cells

**Figure 5:**
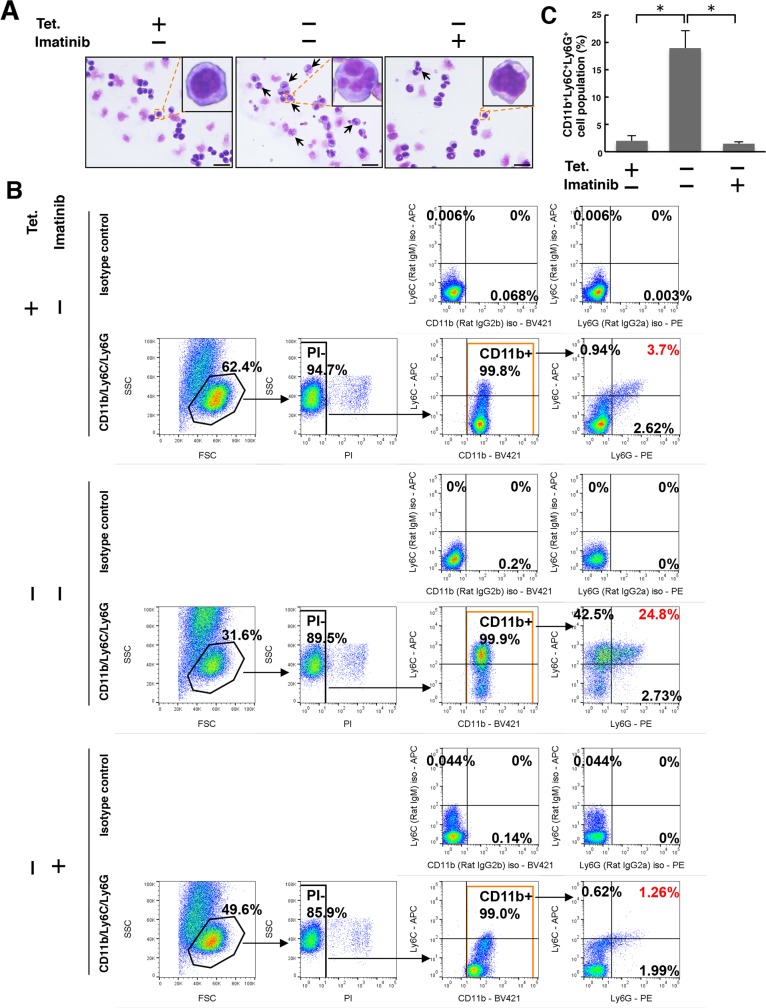
Tet-regulatable overexpression of p210BCR-ABL induces differentiation from myeloid progenitors into G-MDSC in 32D/TetOff-p210 cells 32D/TetOff-p210 cells were Tet-supplied or depleted and then cultured in the presence or absence of imatinib (1 μM) for 96 h. **(A)** Giemsa staining was performed. Cells with segmented nuclei (indicated by arrows) are shown in each panel. Scale bar, 10 μm. **(B)** Cells were triple-stained with anti-CD11b-BV421, anti-Ly6C-APC, and anti-Ly6G-PE, or isotype anti-rat IgG2b-BV421, anti-rat IgM-APC, and anti-rat IgG2a-PE. Stained cells were analyzed by flow cytometry. The obtained data were processed by selection of PI^-^ and CD11b^+^ cell population, and then the cell surface expression of Ly6C and Ly6G within the CD11b^+^ cell population was analyzed. Numbers in the plots indicate the percentages of gated cells. **(C)** Three independent triple-staining experiments, as exemplified in panel B, were performed, and statistical analysis was executed. ^*^P < 0.01. Data are shown as mean ± SEM (*n* = 3).

Although roughly 40% of the 32D/TetOff-p210 cells showed cell death 96 h after Tet-deprivation (Figure 2C), it is not clear that whether differentiation occurred before cell death. Because 32D cells can differentiate into neutrophils in response to G-CSF stimulation [17] and S100A8 proteins are abundantly expressed in neutrophils [28], we next performed Giemsa staining to examine whether Tet-regulatable overexpression of p210BCR-ABL induced segmentation of nuclei. Tet-regulatable overexpression of p210BCR-ABL by Tet-depletion partially induced the differentiation from myeloid progenitors into neutrophil-like cells, characterized by segmented nuclei (indicated by arrows) in 32D/TetOff-p210 cells, and was almost completely inhibited by treatment with imatinib (Figure 5A). Furthermore, the CD11b^+^Ly6C^+^Ly6G^+^ cell population was specifically increased by 24.8% in Tet-depleted 32D/TetOff-p210 cells compared with that in Tet-supplied one (3.7%), and the increase in the CD11b^+^Ly6C^+^Ly6G^+^ cell population in Tet-depleted 32D/TetOff-p210 cells was significantly inhibited by the presence of imatinib (reduced to 1.26%) (Figure 5B and 5C). Meanwhile, the CD11b^+^Ly6C^+^Ly6G^+^ cell population was only 0.011% in parental 32D cells (Supplementary Figure 5). We examined p210BCR-ABL-induced both cell death and differentiation at both 48 h and 96 h after Tet-depletion in 32D/TetOff-p210 cells. The CD11b^+^Ly6C^+^Ly6G^+^ cell population was only 0.13% at 48 h and it was increased by 7.84% at 96 h (Supplementary Figure 6A). Meanwhile, the PI^+^ cell population was 38.6% at 48 h and it was increased by 58.8% at 96 h (Supplementary Figure 6B). We showed the 100% stacked column chart dividing whole cell population into living cells (CD11b^+^Ly6C^+^Ly6G^+^ cells), living cells (undifferentiated cells), and dead cells (PI^+^ cells) in Tet-depleted 32D/TetOff-p210 cells (Supplementary Figure 6C). These data suggest that the cells survived from p210BCR-ABL expression differentiate into G-MDSC. Although differentiated cells eventually die, we cannot perfectly show evidence that G-MDSC differentiation is pre-requite for cell death. Furthermore, mRNA expression of CCAAT/enhancer binding protein α (C/EBPα), the principal regulator of granulocytic differentiation, was detected in both Tet-supplied or Tet-depleted 32D/TetOff-p210 cells, and *Cebpa* mRNA expression in Tet-depleted cells was significantly inhibited in the presence of imatinib (Supplementary Figure 7, left). Our data suggest that Tet-regulatable overexpression of p210BCR-ABL induces differentiation from myeloid progenitor cells into G-MDSC in 32D/TetOff-p210 cells.

### *In vivo* analysis of differentiation into G-MDSC in a CML murine model

**Figure 6:**
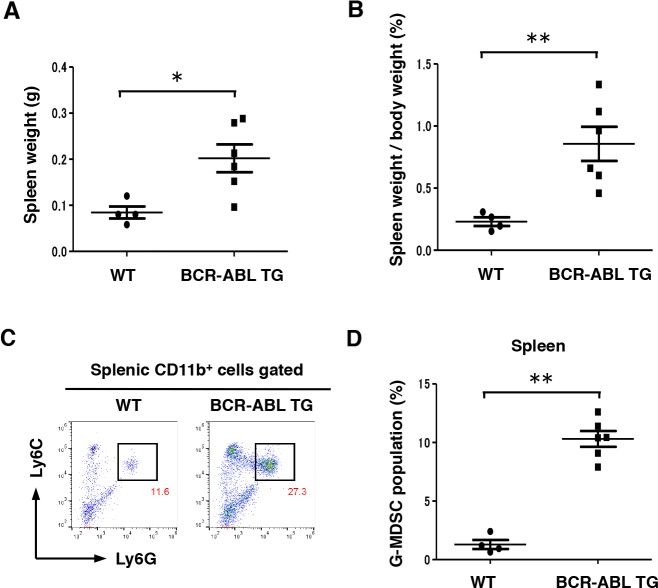
Differentiation into G-MDSC was enhanced in the spleen of BCR-ABL TG mice **(A** and **B)** Absolute spleen weight and relative spleen weight to the whole-body weight of 8-month-old male WT (*n* = 4) and BCR-ABL TG (*n* = 6) mice. Data are shown as mean ± SEM.^*^P < 0.05,^**^P < 0.01. **(C)** Representative plots of G-MDSCs (Ly6C^+^ and Ly6G^+^ cells after gating on CD11b^+^ cells) in the spleen of 8-month-old male WT and BCR-ABL TG mice. Numbers in the plots indicate the percentages of gated cells. **(D)** Relativeratios of G-MDSCs in the spleen of 8-month-old male WT (*n* = 4) and BCR-ABL TG (*n* = 6) mice. Data are shown as mean ± SEM. ^**^P < 0.01.

To examine G-MDSC differentiation *in vivo* in a CML murine model, we collected cells from the spleen of BCR-ABL TG and WT mice. Both spleen and relative spleen weight increased in 8-month-old male BCR-ABL TG mice compared with those in WT mice (Figure 6A and 6B). According to flow cytometric analysis, G-MDSC population (CD11b^+^Ly6C^+^Ly6G^+^) significantly increased in the spleen of BCR-ABL TG mice compared with that in WT mice (Figure 6C and 6D). Individual data of flow cytometric analysis (BCR-ABL TG; *n* = 6, WT; *n* = 4) are also shown in Supplementary Figure 8. These data suggest that increased numbers of splenic G-MDSC may contribute to immunosuppression following the development of CML in the TG model.

## DISCUSSION

In the present study, we clearly revealed that the Tet-regulatable overexpression of p210BCR-ABL induces granulocytic differentiation from myeloid progenitor cells in the 32D/TetOff-p210 cells. Previous studies have shown that G-CSF receptor mRNA is suppressed in v-*abl*-transformed 32Dcl3 [29], and two CML cell lines, K562 and BV173, do not express G-CSF mRNA [30]. Our findings are not consistent with a previous report in which granulocytic differentiation was inhibited in 32Dcl3 cells stably expressing p210BCR-ABL (32D/p210BCR-ABL cells) caused by transcriptional suppression of G-CSF receptor, possibly through downregulation of C/EBPα [31, 32]. These studies, which are totally different from our experimental system, were performed using 32D/p210BCR-ABL cells, which are insusceptible to G-CSF because of G-CSF receptor is not detected even in the presence of G-CSF. However, our established 32D/TetOff-p210BCR-ABL cells exhibited susceptibility to G-CSF because expressions of both G-CSF receptor (*Csf3r*) and G-CSF (*Csf3*) were significantly induced by Tet-regulatable overexpression of p210BCR-ABL, which was restored by treatment with imatinib (Supplementary Figure 7, left). In neutrophils of patients with CML, p210BCR-ABL levels decrease compared with those in immature CML cells but are still retained [33]. G-CSF receptor expression in neutrophils of patients with CML is also observed [34]. Therefore, we assume that G-CSF signaling via G-CSF receptor may involve granulocytic differentiation in patients with CML.

Although the reported HL-60/Bcr-Abl and K562 cells are in a chronic state, imatinib failed to decrease both differentiation and apoptosis in those cells [35], this phenomenon may be experimentally restricted to 32D/TetOff-p210 cells. Although we showed that K562 cells had a SCAT1 cleavage activity (Figure 1D), a high rate of spontaneous cell death was not observed in K562 cells as compared with Tet-depleted 32D/TetOff-p210 cells (Supplementary Figure 9). Therefore, p210BCR-ABL-induced both cell death and differentiation may be experimentally restricted to 32D/TetOff-p210 cells.

Our findings suggest that Tet-regulatable overexpression of p210BCR-ABL-induced cell death and differentiation may be relevant to the induction of G-MDSC in CML. A recent study has shown that the frequency of G-MDSC is significantly increased in patients with CML compared with that in healthy donors and that CML patient-derived mesenchymal stem cells activate G-MDSC, which express higher levels of arginase-1, TNF-α, and IL-1β mRNA [36]. Several clinical studies have shown that imatinib reduces G-MDSC numbers, which exert immunosuppressive activity linked to arginase-1-induced inhibitory effect on T cells, in patients diagnosed with CML [37, 38]. These reports are partially consistent with our findings. Imatinib reduces the number of progenitor cells from which G-MDSC differentiate, therefore imatinib can probably decrease G-MDSC numbers. We showed that G-MDSC differentiation *per se* is dependent on the tyrosine kinase activity of p210BCR-ABL in an imatinib-sensitive manner. The differentiation is accompanied by C/EBPα upregulation, which is consistent with a previous report [39]. An interesting finding is the upregulation of S100A8/A9 that is responsible for activation of MDSC [40], which was also demonstrated in our CML mice model (Figure 4C and 4D). Although we have not tested if sorted G-MDSC from BCR-ABL TG mice are susceptible to cytotoxic drugs including imatinib, those cells express both TLR4 and S100A8 [25] can activate NF-κB [41] in those cells possibly giving resistance against cell death.

Because a previous study has shown that the nuclear entrapment of p210BCR-ABL induces apoptosis of CML cells [42], we hypothesized that p210BCR-ABL-induced cell death observed in 32D/TetOff-p210 cells may be caused by the nuclear entrapment of p210BCR-ABL. To test this hypothesis, we examined the cellular localization of p210BCR-ABL in 32D/TetOff-p210 cells. Immunofluorescent staining using anti-Abl antibody showed that Tet-depletion-induced p210BCR-ABL proteins were predominantly localized in the cytosol but not in the nucleus (Supplementary Figure 10). Imatinib did not affect the localization of p210BCR-ABL (Supplementary Figure 10). These results suggest that p210BCR-ABL-induced cell death is not caused by the nuclear entrapment of p210BCR-ABL in 32D/TetOff-p210 cells.

Imatinib is an effective drug for targeting chronic phase CML. However, imatinib resistance is a problem, particularly in the advanced CML phase. Resistance mechanisms are currently explained by additional genetic mutation, natural imatinib resistance in CML stem cells, and overexpression of BCR-ABL [2, 16, 43]. Our results may provide insights into the overexpression-related resistance. Our data suggest that a paradoxical counteraction of imatinib against cell survival exists in CML. However, it may be possible that imatinib exerts different effects between proliferating cells and differentiating cells in CML, meaning that imatinib exerts growth-inhibitory effects against proliferating cells, as exemplified in 32D/p210BCR-ABL cells, and differentiation-suppressive effects against differentiating cells, as exemplified in Tet-regulatable p210BCR-ABL-expressing 32D/TetOff-p210 cells. A clinical report has suggested synergistic effects of imatinib and G-CSF against CML [44].

In the present study, we propose a novel mode of cell death, proposed as myeloptosis, which has combined features of caspase-1-induced pyroptosis and caspase-3-induced apoptosis, coincident with the differentiation from myeloid progenitors into G-MDSC and the secretion of IL-1β, TNF-α, and S100A8/A9 induced by Tet-regulatable overexpression of p210BCR-ABL in 32D/TetOff-p210 cells. Furthermore, increased numbers of G-MDSC due to enhancement of S100A8/A9 production were observed in TG mice expressing p210BCR-ABL. Myeloptosis may create a vicious cycle comprising a continuous loop of cell death-induced secretion of proinflammatory cytokines and accumulation of G-MDSC in CML possibly responsible for immunosuppression.

## MATERIALS AND METHODS

### Materials

Antibody against Flag tag and CHX were purchased from Sigma-Aldrich (St Louis, MO, USA). Antibody against Myc tag was purchased from Life technologies (Rockville, MD, USA). Antibody against actin and IM-54 was purchased from Merck Millipore (Billerica, MA, USA). Antibodies against Abl (sc-23), phospho Tyr (PY99, sc-7020), caspase-1 p10 (sc-514), S100A8 (sc-8113), S100A9 (sc-8115) and caspase-1 inhibitor (z-YVAD-fmk) were purchased from Santa Cruz Biotechnology (Santa Cruz, CA, USA). Antibodies against cleaved caspase-3 and cleaved PARP were purchased from Cell Signaling Technology (Danvers, MA, USA). Blocking antibody against mouse TNF and purified NA/LE hamster IgG1, λ1 isotype control was purchased from BD Biosciences (San Jose, CA, USA). Blocking antibody against mouse IL-1β was purchased from R&D systems. (Minneapolis, MN, USA). Imatinib mesylate was purchased from Novartis (Basel, Switzerland). Human TNF-α was purchased from Peprotech (Rocky Hill, NJ, USA). Q-VD-OPH was purchased from TONBO biosciences (San Diego, CA, USA). Glibenclamide was purchased from Wako (Osaka, Japan).

### Plasmids

SCAT1 cDNA, which was subcloned in-frame into pcDNA3.1 vector (Life technologies), was kindly donated from Dr. Yoshifumi Yamaguchi and Dr. Masayuki Miura. p210BCR-ABL and p210BCR-ABL kinase dead (KD, ABL K290R) mutant cDNA were subcloned in-frame into pFlag-CMV2 vector (Sigma-Aldrich). Tet-regulatable p210BCR-ABL construct, pUHD 10-3 BCR-ABL IRES GFP, and Tet-regulated transactivator (tTA) expression vector, pCAG20-1, were kindly donated from Dr. Owen N. Witte.

### Animal experiments

p210*^bcr/abl^* TG (*BCR-ABL^tg/-^*; BCR-ABL TG) mice were generated as previously described [27]. The normal littermates of these mice (*bcr/-abl^-/-^*) were used as controls. Mice at 8 months of age that already exhibited a CML-like phenotype were sacrificed, and spleen cells were collected by a standard procedure. Experimental procedures performed on mice were approved by the Animal Research Committee of Tokyo Women's Medical University (AE17-59).

### Cell culture

The human cervical carcinoma cell line HeLa was cultured in DMEM with 10%(v/v) FBS at 37°C under 5% CO2 in air. The human CML cell line K562 was cultured in RPMI1640 with 10%(v/v) FBS at 37°C under 5% CO2 in air. Mouse myeloid progenitor 32D cells were pharchased from RIKEN BioResouce Centaer Cell Bank (RBRC-RCB1145; Tsukuba, Ibaraki, Japan) and cultured in RPMI1640 supplemented with 10%(v/v) FBS and 10%(v/v) IL-3 culture supplement (Corning, NY, USA), defined as the 32D growth media, at 37°C under 5% CO2 in air. 32D/TetOff-p210 cells were maintained in 32D growth media supplemented with Tet (1 μg/ml) without IL-3 culture supplement. For Tet-depletion, cultured cells were washed two times with RPMI1640 supplemented with 10%(v/v) FBS and were then cultured in 32D growth media without Tet. Ba/F3 cells stably expressing Flag-tagged p210BCR-ABL were cultured in RPMI1640 supplemented with 10%(v/v) FBS. All cell lines using in the present study were mycoplasma-negative certificated by using MycoAlert™ Mycoplasma Detection Kit (Lonza, Allendale, NJ, USA).

### Transfection

Transfection was performed by electroporation using an Amaxa CLB transfection reagent (Lonza), according to the manufacturer's protocol.

### Immunoblotting

WCL were prepared by cell lysis with 50 mM Tris-HCl (pH 7.4)/0.1%SDS/0.5%NP-40, containing protease inhibitor mixtures, on ice for 30 min. Next, the supernatant was collected after centrifugation (15,000 rpm for 10 minutes). The conditioned medium was prepared by collecting the supernatant after centrifugation (1,500 rpm for 5 minutes) and condensed by trichloroacetic acid precipitation. The samples were mixed with a SDS sample buffer containing 2-mercaptoethanol and boiled for 3 min, electrophoresed on precast gradient gels (Wako, SuperSep™ Ace, 5-20% or 10-20%), and electro-transferred using a Trans-Blot-Turbo transfer system (Bio-rad, Hercules, CA, USA) to a polyvinylidene difluoride membrane (Bio-Rad). The membranes were blocked with TBS-0.1% (v/v) Tween 20 (TBS-T) with 0.5% (w/v) non-fat milk, and were incubated with a primary antibody overnight, followed by TBS-T washes and incubation with a horseradish peroxidase-conjugated secondary antibody for 30 min. Protein bands were developed using Pierce Western Blotting Substrate (Thermo Fisher Scientific, Waltham, MA, USA).

### WST-8 assay

Cell viability was determined by colorimetric cell proliferation assay using WST-8. WST-8 is a non-cell permeable compound, which is reduced by mitochondrion dehydrogenase to form the water-soluble formazan dye that is directly proportional to viable cells. 32D/TetOff-p210 or parental 32D cells (1 × 10^4^ cells) were Tet-supplied or depleted and then cultured with IL-3 supplement in the presence or absence of imatinib (1 μM) for 48 h in a 96-well microplate. Cell counting kit-8 reagent (DOJINDO LABORATORIES, Kumamoto, Japan) was added to each well and then incubated at 37°C for 4 h. After incubation, absorbans at 450 nm was measured using Multiscan™GO Microplate Spectrophotometer (Thermo Fisher Scientific) to determine cell viability.

### Immunofluorescence microscopy

Cultured cells were mounted with Smear Gell (GenoStaff, Tokyo, Japan) on APS-coated slide glass (Matsunami glass, Osaka, Japan) and fixed with 4%(w/v) paraformaldehyde/phosphate buffered saline (PBS) (Wako) for 20 minutes. After fixing, cells were washed with PBS and incubated with 0.1%(v/v) Triton X-100 for 5 min. After blocking in Blocking One Histo (Nakarai tesque, Kyoto, Japan) for 30 min at room temperature, a primary antibody against Abl was added to the cells. The cells were then washed three times with PBS and incubated with Alexa Fluor 555 labeled anti-mouse IgG antibody (Life technologies). The images were obtained using a confocal laser scanning microscope (LSM510 META, Carl Zeiss) and processed using Zeiss LSM Image Browser version 4.2.

### Flow cytometry

Double staining of annexin V and PI was performed using Annexin V Apoptosis detection set phycoerythrin (PE)-Cy7 (eBioscience, San Diego, CA, USA) or Brilliant Violet 421™ (BV421) Annexin V (BioLegend, San Diego, CA, USA), and activated caspases detection was performed using a FLICA 660 caspase-1 or caspase-3/-7 assay kit (Immunochemistry Technologies, Bloomington, MN, USA) according to the manufacturer's protocol. Stained cells were analyzed with a flow cytometer Cytomics FC 500 (Beckman Coulter, Fullerton, CA, USA). Triple staining of CD11b, Ly6C, and Ly6G for 32D/TetOff-p210 cells was performed using anti-CD11b-BV421, anti-Ly6C-allophycocyanin (APC) (BD Biosciences), and anti-Ly6G-PE (BD Biosciences), respectively. Stained cells were analyzed with a high-speed cell sorter MoFlo Astrios^EQ^ (Beckman Coulter, Fullerton, CA, USA). Triple staining of CD11b, Ly6C, and Ly6G for spleen cells from mice was performed using anti-CD11b-PE (BD Biosciences) anti-Ly6C-APC (BD Biosciences), and anti-Ly6G-fluorescein isothiocyanate (FITC) (BD Biosciences), respectively. Stained cells were analyzed with a flow cytometer Cytomics FC 500 (Beckman Coulter).

### Giemsa staining

32D/TetOff-p210 cells were Tet-supplied or depleted and then cultured in the presence or absence of imatinib (1 μM) for 96 h. Next, a smear was prepared on a slide glass and fixed with 100% methanol for 2 min. After fixing, Giemsa staining solution diluted using M/150 phosphate buffer (pH6.4) was added and incubated for 20 min. After washing with distilled water, the prepared slide was allowed to air-dry and was visualized under the microscope.

### ELISA

The concentration of mouse IL-1β and TNF-α in the cell culture supernatants was determined using the Quantikine ELISA Kit (R&D systems) according to the manufacturer's protocol. The concentration of mouse S100A8/A9 complex in the cell culture supernatants was determined using S100A8/A9 ELISA kit (Immundiagnostik AG, Bensheim, Germany) according to the manufacturer's protocol.

### Quantitative RT-PCR analysis

Total RNA was isolated from cells using ISOGEN II (Nippon Gene, Tokyo, Japan). cDNA was synthesized using PrimeScript™ II with an Oligo(dT)_15_ Primer (Takara Bio, Shiga, Japan). Quantitative PCR analysis was performed using the SYBR^®^ Premix Ex Taq II (Takara Bio) and then quantified using StepOnePlus™ Real Time PCR system (Applied Biosystems, Foster City, CA, USA). For *S100a8* and *S100a9* analysis, RNA was reverse transcribed and quantitative PCR analysis was performed using SYBR Green master mixture (TOYOBO) and ABI 7500 Sequence Detection System (Applied Biosystems). Gene level was calculated from cycle threshold values, and a logarithm of the copy number of a target gene was confirmed to be on a linear line using the corresponding isolated DNA and its serial dilutions as a standard. Thus, gene level for each target mRNA was normalized against that of β-actin (*Actb*) for each sample. Both *S100a8* and *S100a9* mRNA were normalized against that of glyceraldehyde-3-phosphate dehydrogenase (*Gapdh*) in each sample. The following primers were used: *S100a8*, 5′-CCG TCT TCA AGA CAT CGT TTG A-3′ and 5′-GTA GAG GGC ATG GTG ATT TCC T-3′; *S100a9*, 5′-GTC CAG GTC CTC CAT GAT GT-3′ and 5′-GAA GGA AGG ACA CCC TGA CA-3′; *Cebpa*, 5′-CAA GAA GTC GGT GGA CAA GAA-3′ and 5′-CGT TGC GTT GTT TGG CTT TA-3′; *Csf3r*, 5′-GGG ACC TCT TCA CCT ACT ACA-3′ and 5′-CAG TCT ACC CAG ATG GTG TTA AG-3′; *Csf31*, 5′-TCC TGC TTA AGT CCC TGG AG-3′ and 5′-TGA CAC AGC TTG TAG GTG GC-3′; *Actb*, 5′-TTC TTT GCA GCT CCT TCG TT-3′ and 5′-ATG GAG GGG AAT ACA GCC C-3′.

### Statistical analysis

Data are expressed as the means ± standard error of the mean (SEM). The significance of differences was analyzed using one-way ANOVA and Bonferroni test or Mann–Whitney *U* test. *P* < 0.05 was considered significantly different.

## SUPPLEMENTARY MATERIALS FIGURES



## Abbreviations

CML: chronic myeloid leukemia
Tet: tetracycline
G-MDSC: granulocytic myeloid-derived suppressor cells
M-MDSC: monocytic myeloid-derived suppressor cells
IL-1β: interleukin-1β
TNF-α: tumor necrosis factor-α
FRET: fluorescence resonance energy transfer
NLRP3: Nod-like receptor family
SCAT1: a sensor of activated caspase-1 based on FRET
TG: transgenic
WT: wild-type
CHX: cycloheximide
WCL: whole cell lysate
G-CSF: granulocyte-colony stimulation factor
WST-8: [2-(2-methoxy-4-nitrophenyl)-3-(4-nitrophenyl)-5-(2
PI3K: phosphatidylinositol 3 kinase
STAT5: signal transducer and activator of transcription 5
PI: propidium iodide
PARP: poly ADP ribose polymerase
FLICA: fluorescently labelled inhibitor of caspases
ROS: reactive oxygen species
NF-κB: nuclear factor-κB
C/EBPα: CCAAT/enhancer binding protein α
PE: phycoerythrin
APC: allophycocyanin
FITC: fluorescein isothiocyanate
