# Supplementary material for: Paradoxical counteraction by imatinib against cell death in myeloid progenitor 32D cells expressing p210BCR-ABL
Source: Oncotarget. 2018 Aug 3;9(60):31682–96. doi: 10.18632/oncotarget.25849 (PMC6114964; doi:10.18632/oncotarget.25849)
Supplement: Supplementary file 1 [file oncotarget-09-31682-s001.pdf]

Paradoxical counteraction by imatinib against cell death in myeloid progenitor 32D cells expressing p210BCR-ABL

SUPPLEMENTARY MATERIALS

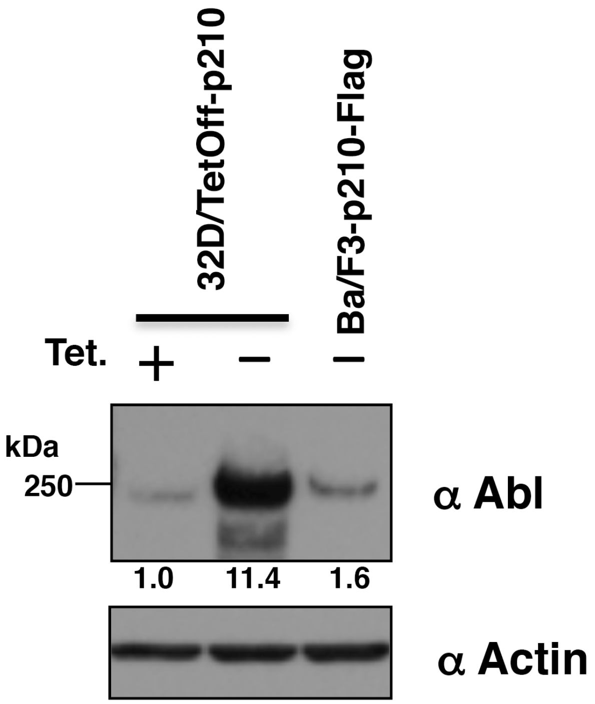

Supplementary Figure 1: Comparison of expression level of p210BCR-ABL protein between 32D/TetOff-p210 and Ba/F3-p210-Flag cells. 32D/TetOff-p210 cells were Tet-supplied or depleted and then cultured for 48 h. Flag-tagged p210BCR-ABL (p210-Flag)-expressing stable Ba/F3 cells were cultured. WCL were subjected to immunoblotting. The values of relative band intensity versus 32D/TetOff-p210 Tet (+) control were shown below panel.

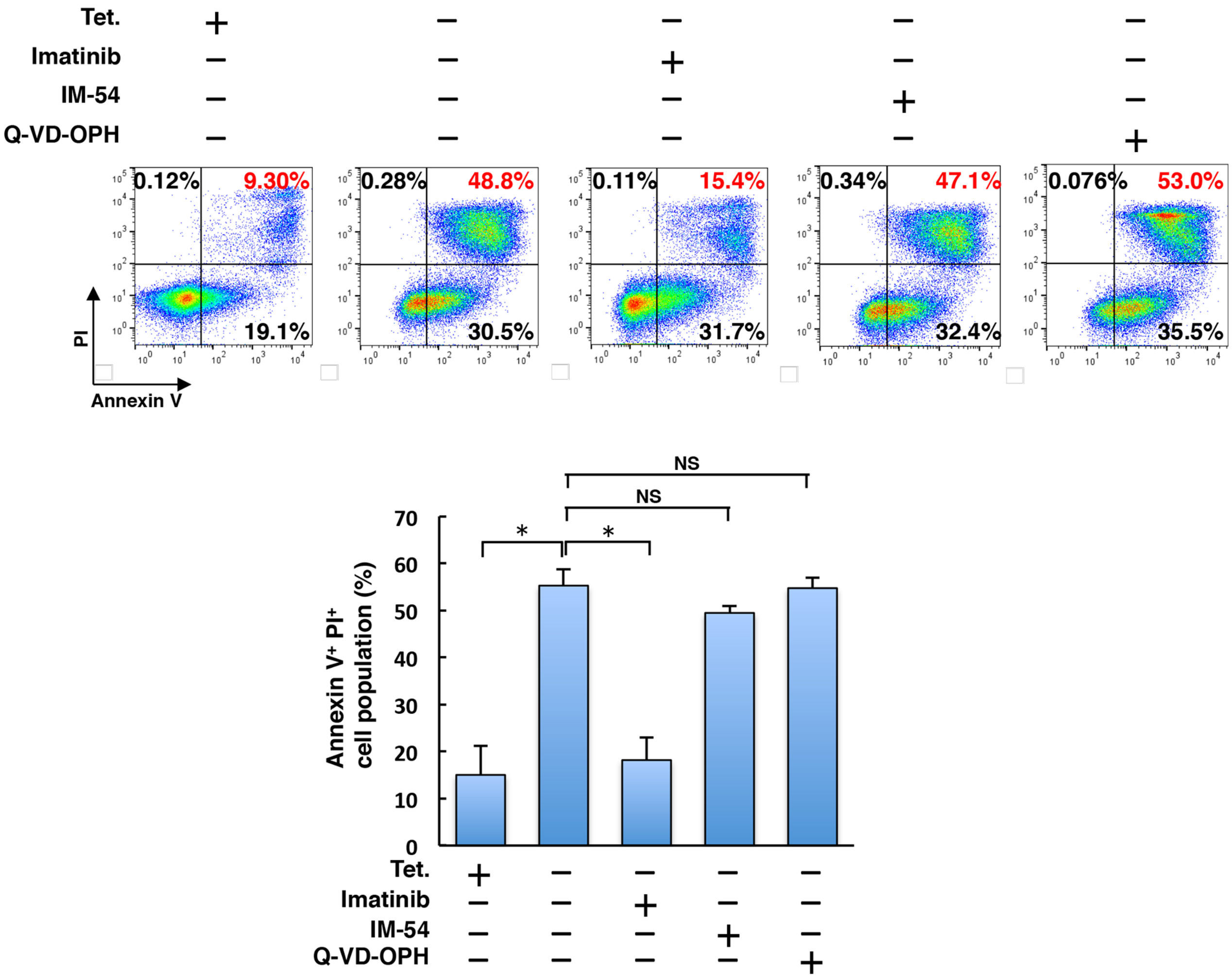

Supplementary Figure 2: Cell death analysis of 32D/TetOfff-p210 cells in the presence of the different cell death inhibitors. 32D/TetOff-p210 cells were Tet-supplied or depleted and then cultured for 96 h in the presence or absence of imatinib (1 μM), IM-54 (3 μM), Q-VD-OPH (20 μM). Cells were double-stained with annexin V-BV421 and PI and analyzed by flow cytometry. The proportion of cell population with the representative data is shown in each panel. Three independent double-staining experiments were performed and statistical analysis was executed. $^{*}P < 0.001$. Data are shown as mean ± SEM ($n = 3$). NS indicates no significant difference.

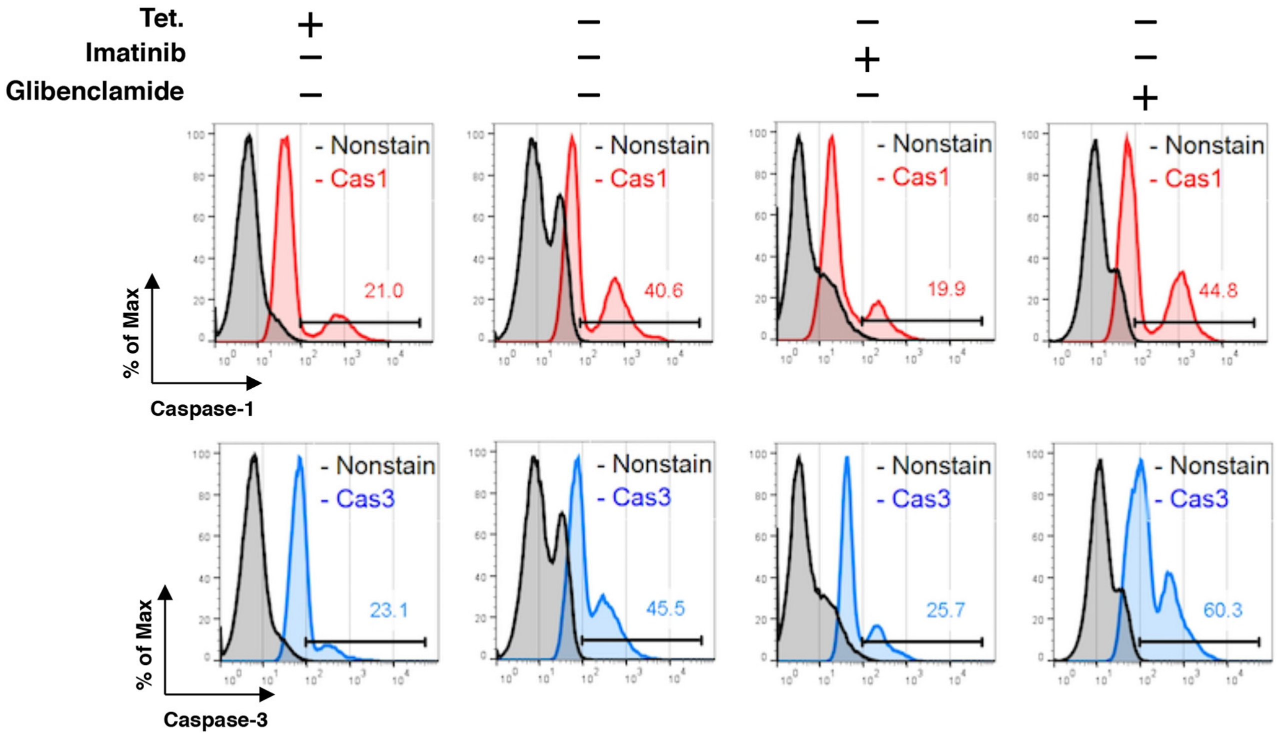

Supplementary Figure 3: Flow cytometric analysis of the activation of both caspase-1 and caspase-3 in 32D/TetOff-p210 cells in the presence of a NLRP3 inhibitor. 32D/TetOff-p210 cells were Tet-depleted or supplied and then cultured in the presence or absence of imatinib (1 μM) or glibenclamide (25 μg/ml) for 96 h and then incubated with FLICA 660 active caspase-1 or caspase-3 detection probe and analyzed by flow cytometry. The merged histogram (red or blue) with non-stain control (gray) is shown in each panel. The proportion of FLICA-positive cell population max is shown in each panel.

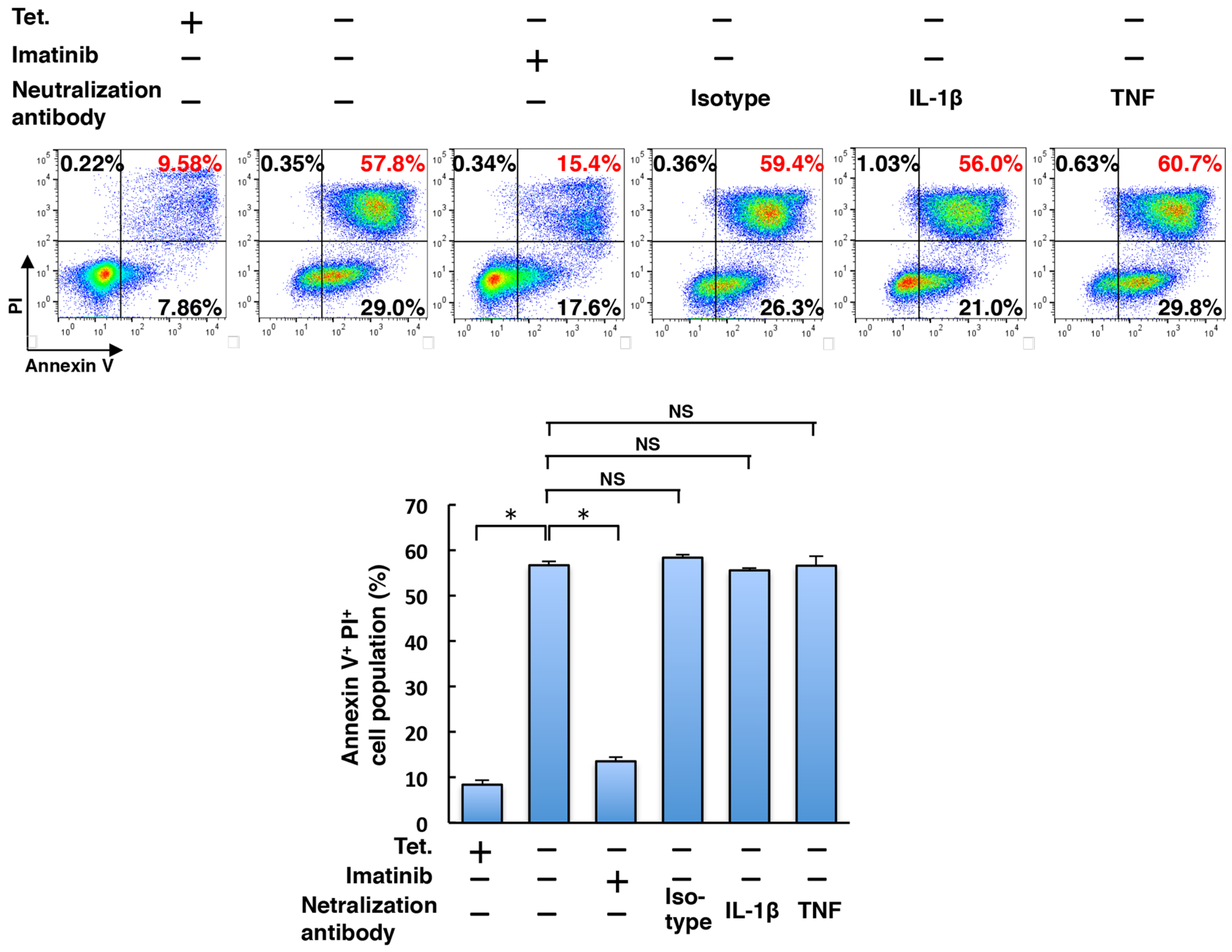

Supplementary Figure 4: Cell death analysis of 32D/TetOfff-p210 cells in the presence of different cytokine blocking antibodies. 32D/TetOff-p210 cells were Tet-supplied or depleted and then cultured for 96 h in the presence or absence of imatinib (1 µM), isotype control antibody (1 µg/ml), anti-IL-1β antibody (1 µg/ml), anti-TNF antibody (1 µg/ml). Cells were double-stained with annexin V-BV421 and PI and analyzed by flow cytometry. The proportion of cell population with the representative data is shown in each panel. Three independent double-staining experiments were performed and statistical analysis was executed. $^{*}P < 0.001$. Data are shown as mean ± SEM ($n = 3$). NS indicates no significant difference.

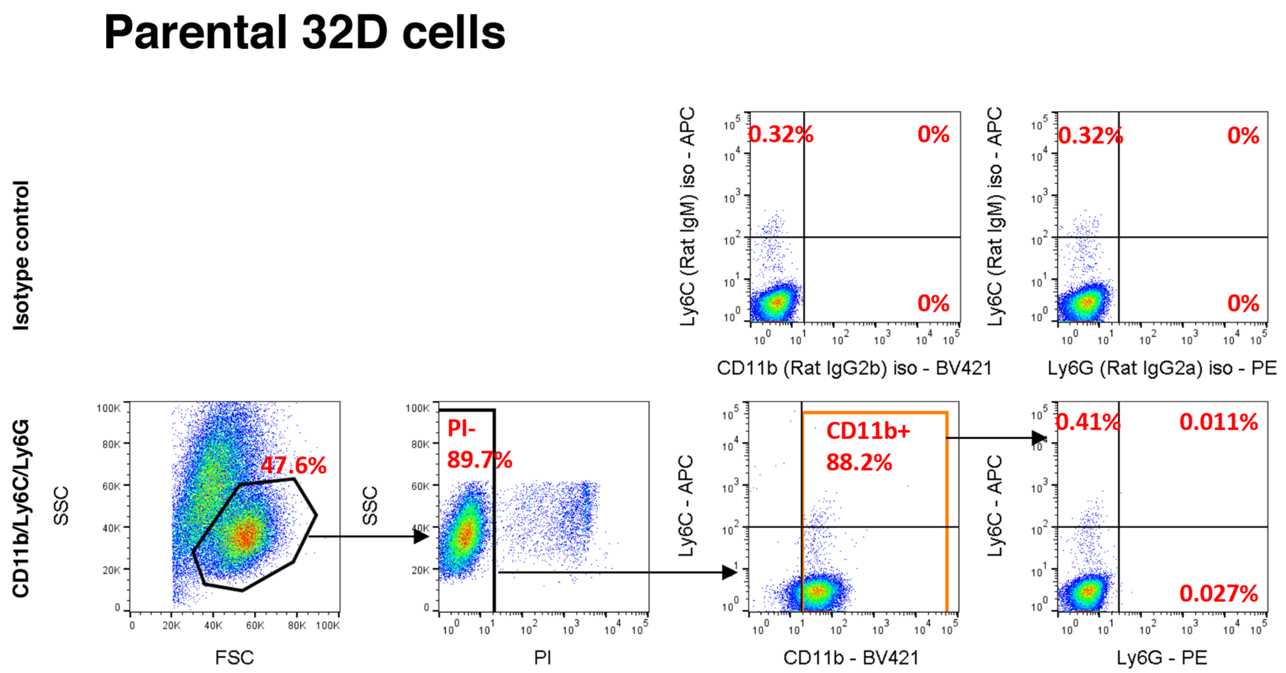

Supplementary Figure 5: Flow cytometric analysis of G-MDSC of parental 32D myeloid progenitor cells. 32D cells were cultured in the presence of IL-3 for 96 h. Cells were triple-stained with anti-CD11b-BV421, anti-Ly6C-APC, and anti-Ly6G-PE, or isotype anti-rat IgG2b-BV421, anti-rat IgM-APC, and anti-rat IgG2a-PE. Stained cells were analyzed by flow cytometry. The obtained data were processed by selection of PI^- and $CD11b^+$ cell population, and then the cell surface expression of Ly6C and Ly6G within the $CD11b^+$ cell population was analyzed. Numbers in the plots indicate the percentages of gated cells. Representative data is shown in three independent experiments.

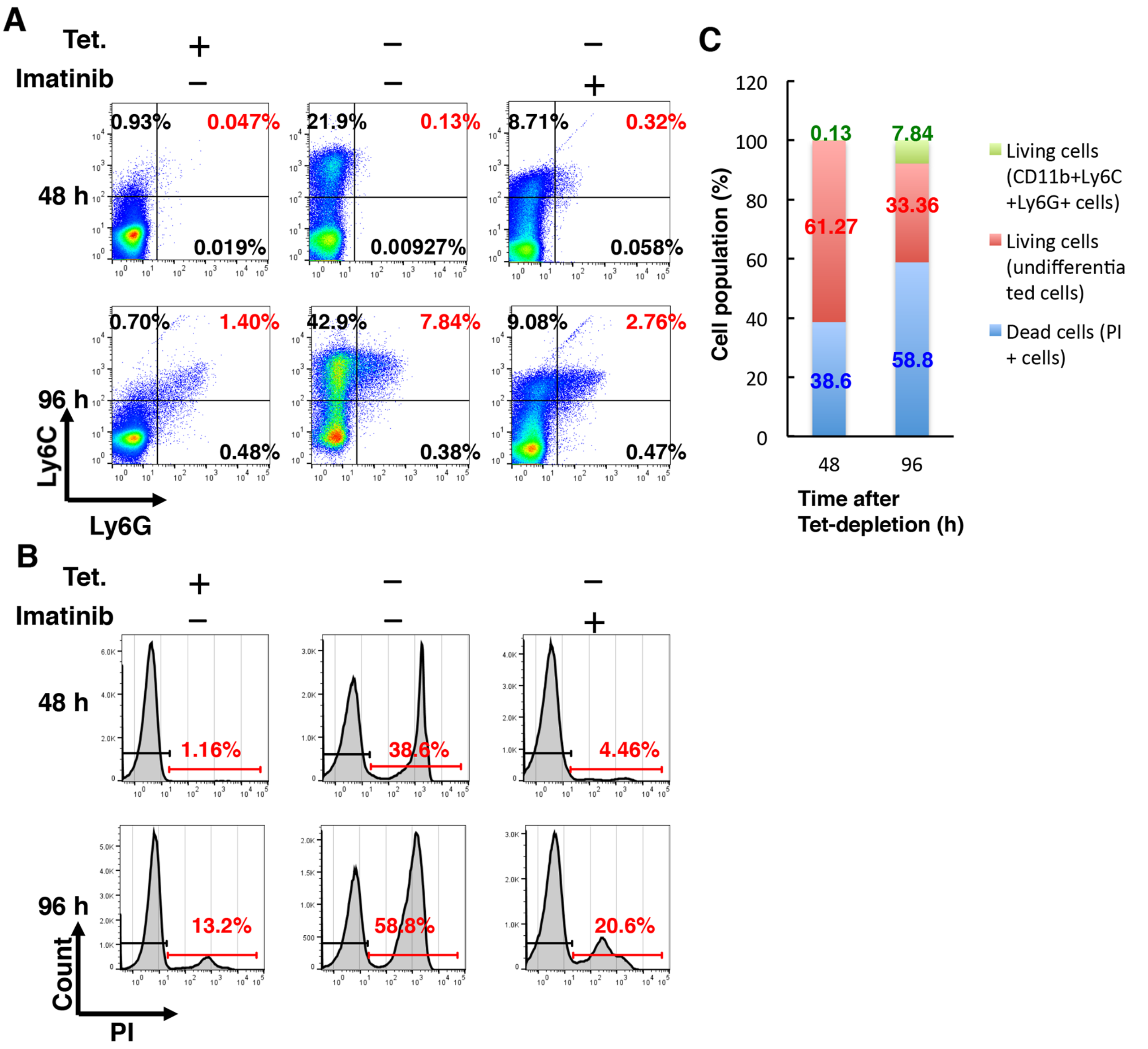

Supplementary Figure 6: Flow cytometric analysis of both G-MDSC differentiation and cell death in 32D/TetOff-p210 cells. 32D/TetOff-p210 cells were Tet-depleted or supplied and then cultured in the presence or absence of imatinib (1 µM) for 48 h or 96 h. **(A)** Cells were triple-stained with anti-CD11b-BV421, anti-Ly6C-APC, and anti-Ly6G-PE, or isotype anti-rat IgG2b-BV421, anti-rat IgM-APC, and anti-rat IgG2a-PE. Stained cells were analyzed by flow cytometry. The obtained data were processed by selection of PI^- and $CD11b^+$ cell population, and then the cell surface expression of Ly6C and Ly6G within $CD11b^+$ cell population was analyzed. Numbers in the plots indicate the percentages of gated cells. **(B)** Cells were stained with PI. The proportion of PI-positive cell population is shown in each panel. **(C)** The 100% stacked column chart with the percentage of each cell population in Tet-depleted 32D/TetOff-p210 cells is shown.

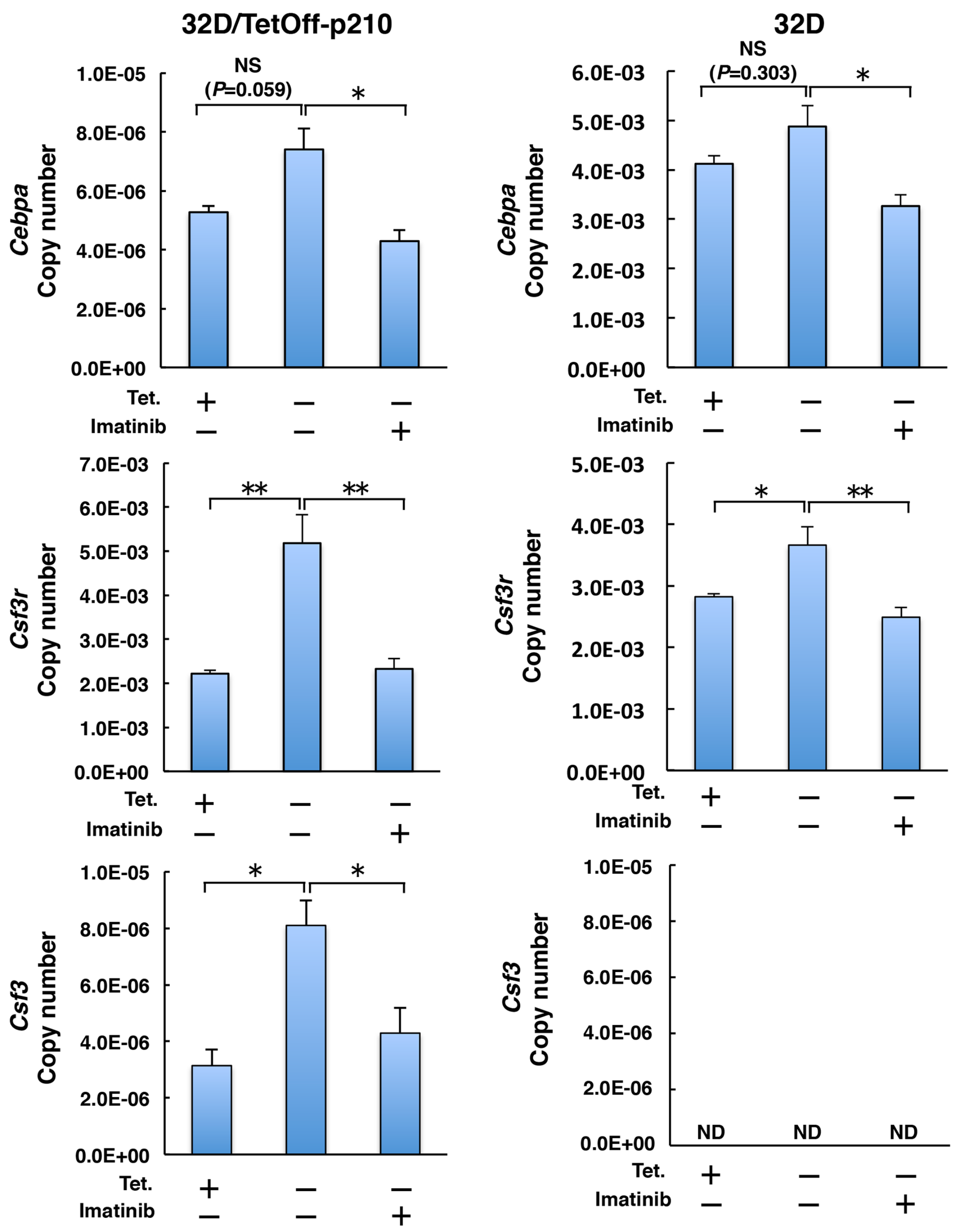

Supplementary Figure 7: Tet-regulatable overexpression of p210BCR-ABL induces expression of both *Csf3r* and *Csf3* mRNA in 32D/TetOff-p210 cells. 32D/TetOff-p210 cells or parental 32D cells were Tet-supplied or depleted and then cultured in the presence or absence of imatinib (1 μM) for 96 h, and then total RNA was isolated from cells and subjected to quantitative RT-PCR analysis to quantify *Cebpa*, *Csf3r, and Csf3* mRNA. $^{*}P < 0.05$, $^{**}P < 0.01$. NS indicates no significant difference. ND indicates not detected (under the detection limit). Data are shown as mean ± SEM (32D/TetOff-p210; $n = 3$, 32D; $n = 4$).

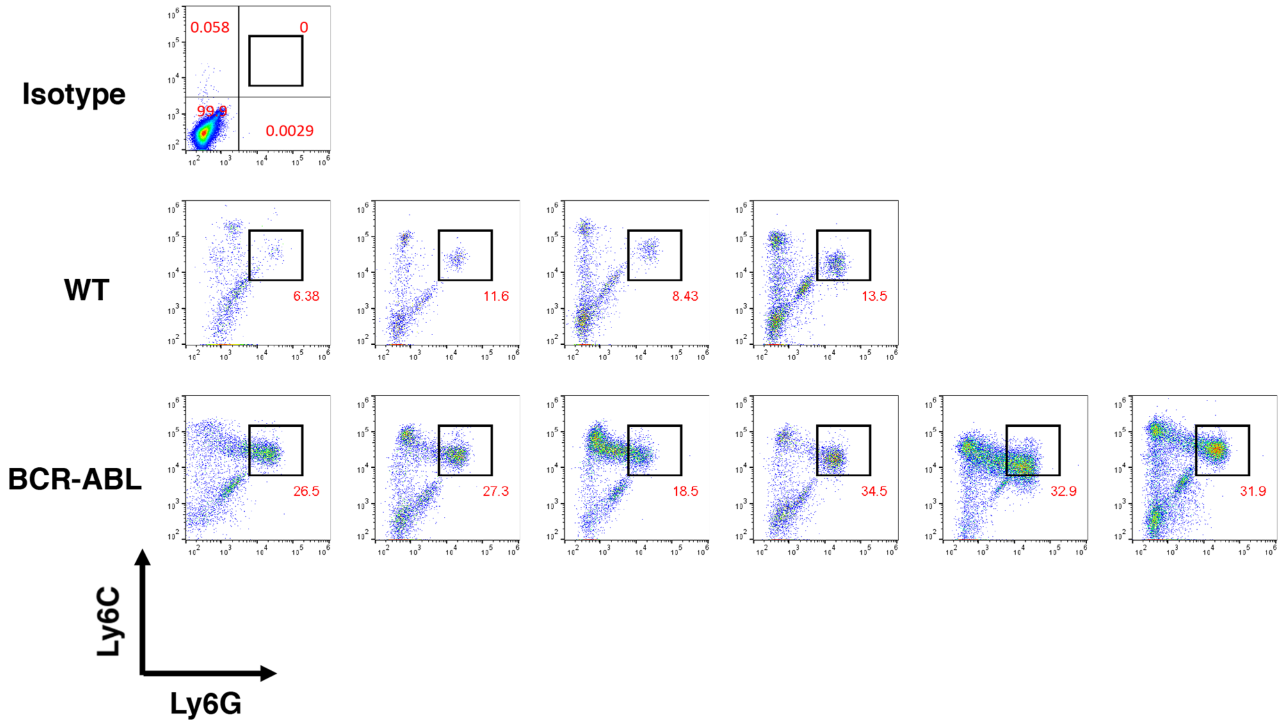

Supplementary Figure 8: Flow cytometric analysis of splenic G-MDSC in BCR-ABL TG mice. Flow cytometry analysis of G-MDSC (Ly6C$^+$ and Ly6G$^+$ cells after gating on CD11b$^+$ cells) in the spleen of 8-month-old male WT (*n* = 4) or BCR-ABL TG (*n* = 6) mice. Numbers in the plots indicate the percent of gated cells.

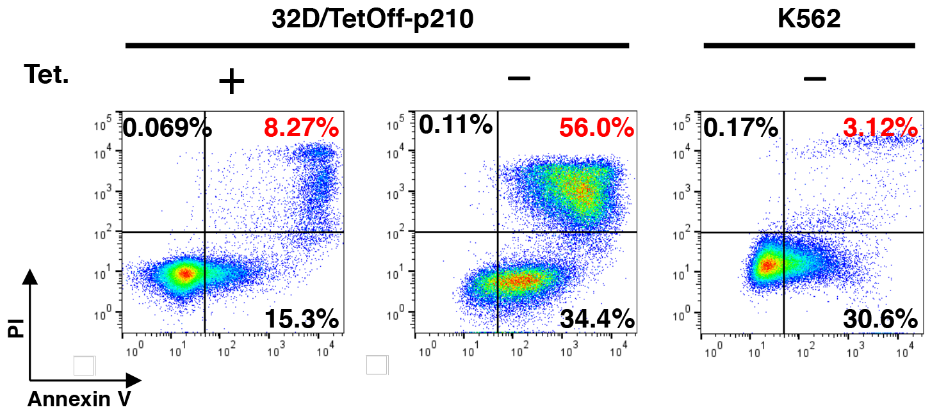

Supplementary Figure 9: Cell death analysis of human leukemic K562 cells. 32D/TetOff-p210 cells were Tet-supplied or depleted and then cultured for 96 h. Human CML cell line K562 cells were cultured for 96 h. Cells were double-stained with annexin V-BV421 and PI and analyzed by flow cytometry. The proportion of cell population with the representative data is shown in each panel.

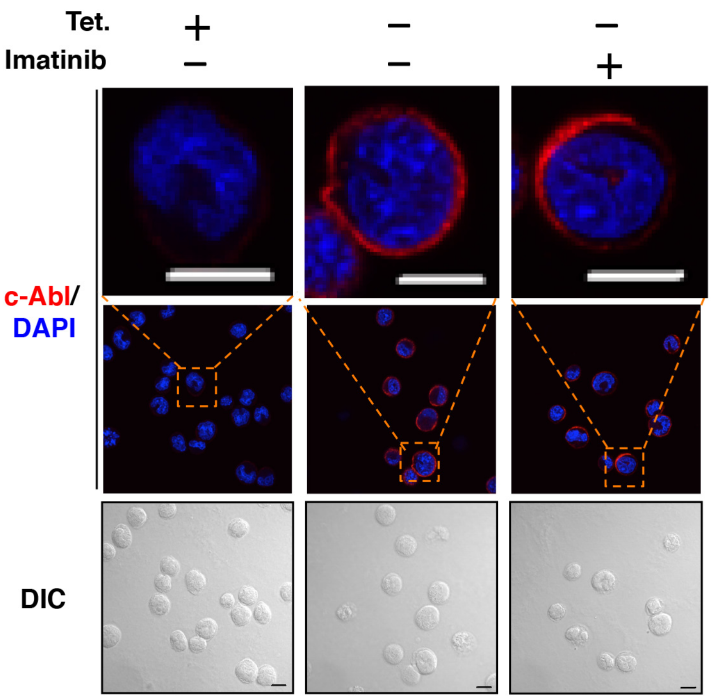

Supplementary Figure 10: Localization of p210BCR-ABL protein in 32D/TetOff-p210 cells. 32D/TetOff-p210 cells were Tet-supplied or depleted and then cultured for 48 h in the presence or absence of imatinib (1 μM). Cells were mounted on slide glass, and immunofluorescent staining was performed. The merged images of anti-Abl (Alexa Fluor 555) and DAPI (4, 6-diamino-2-phenylindole) are shown in upper and middle panels and the images of DIC (differential interference contrast) are shown in lower panels. Scale bar, 10 μm.
